# Anticoagulants Used in Cardiac Catheterization of Patients With Chronic Lymphocytic Leukemia: A Case Report and Overview

**DOI:** 10.7759/cureus.13633

**Published:** 2021-03-01

**Authors:** Anton Mararenko, Abbas Alshami, Mohammed AlAzzawi, Swapnil V Patel

**Affiliations:** 1 Internal Medicine, Jersey Shore University Medical Center, Neptune, USA; 2 Internal Medicine, Hackensack Meridian Health, Jersey Shore University Medical Center, Neptune, USA

**Keywords:** myocardial infarction, pci, stemi, anticoagulant, bivalirudin, cll

## Abstract

Percutaneous coronary intervention (PCI) is one of the most frequently performed invasive therapeutic procedures and plays a key role in the long-term survival of patients with ischemic heart disease. Over 965,000 angioplasties are performed annually in the United States alone. While the technique and equipment have undergone significant revisions and improvement, the medical community will still benefit from more data and guidance on the optimal selection of mandatory peri-operation anticoagulation in specific, high-risk populations. Many of these procedures are performed on high-risk individuals who have an inherently higher risk of hemorrhage or thrombosis. Unfractionated heparin is the most popular choice in the general population, however, its use carries certain limitations. Here we will describe the use of an uncommonly used anticoagulant in a patient being actively treated for leukemia. We will also discuss the unique properties and benefits of the four most frequently used anticoagulants during a cardiac angioplasty. Our team describes the successful use of bivalirudin during an urgent PCI in a 71-year-old female with eight previous stents that was followed by an uncomplicated recovery period. Our experience contributes to a small, but growing, body of evidence that bivalirudin may be a safe choice to use in lieu of unfractionated heparin in patients with underlying oncological disease. Our patient had several comorbidities that significantly increased their risk of bleeding. We will also review the clinical trials that compared the four most commonly used anticoagulants during cardiac catheterization.

## Introduction

Chronic lymphocytic leukemia (CLL) is a clonal malignancy characterized by the overproduction of B type lymphocytes. The disease course is usually indolent with over 90% of cases occurring after the age of 50 years and a median age of diagnosis being 70 years. On presentation, most patients have a profound leukocytosis with a lymphocytic predominance. Approximately 5-10% of cases are known to transform into a more aggressive, unpredictable form known as Richter transformation. From a cardiac perspective, the biggest concerns stem from the abnormal hematopoiesis that can inadvertently lead to hypercoagulability from increased viscosity or bleeding from worsening anemia and thrombocytopenia [1]. In addition, the cardiotoxic profile of medications used in CLL cannot be ignored, especially ibrutinib. Although our patient did not receive this medication, it is a well established medication known to be pro-arrhythmogenic, particularly in exacerbating atrial fibrillation, and increases the risk of bleeding when used in conjunction with warfarin [2].

Novel therapies have changed the dynamic of CLL treatment and prognosis. In the past, median survival was 6 years with only 25% of patients living more than 10 years. Now, patients that start treatment at Stage 0/I as per the Rai system, survival rates are cited to be as high as 10-15 years with an unchanged quality of life. However, while emerging chemotherapeutic agents have changed the dynamic of CLL treatment, we must also consider the interplay with CLL and challenges in treating other comorbidities as well in setting of CLL. As previously mentioned, patients with CLL have a higher risk of hematologic dyscrasias since they can have a high risk of clotting and bleeding. This poses a significant logistical challenge in patients that require invasive surgeries or procedures such as cardiac catheterization.

Percutaneous coronary intervention (PCI) is one of the most frequently performed invasive therapeutic procedures and plays a key role in the long-term survival of patients with ischemic heart disease. While the technique and equipment have undergone significant revisions and improvement, we still need more data and guidance on the optimal selection of anticoagulation in specific, high-risk populations. Currently, the four most studied, and commonly used agents are unfractionated heparin (UF-Heparin), low molecular weight heparin (LMWH), bivalirudin and fondaparinux. According to the ACTION registry that reviewed 66,000 patients who underwent PCI from 2007-2010, UF-Heparin was the most used agent in 80% of patients followed by 23% receiving bivalirudin and 11% receiving LMWH PCI [3]. According to the European and US-AHA guidelines for acute ST elevation myocardial infarction (STEMI), the three most viable options include heparin with glycoprotein (GPI), bivalirudin alone or heparin alone - all of which are Class I or Class IIA recommendations [4-5]. As bleeding is an inherent risk when using anticoagulants in any population, we must give extra consideration when selecting an anticoagulant in patients predisposed to bleeding due to comorbidities. The analysis performed by Mamas et al. encompasses a nationwide analysis on the in-hospital outcomes of patients that underwent PCI. They reviewed that 10,800 patients with a diagnosis of CLL underwent PCI from 2004-2014 and were found to have a significantly increased risk of bleeding, as well as mortality risk [6].

It remains unclear which agent is best in patients with comorbidities that increase their risk of bleeding. We present a case of successful use of bivalirudin during PCI of a patient with CLL and eight previous stents. They were administered bivalirudin during PCI without complication despite bivalirudin being associated with a higher risk of stent thrombosis.

## Case presentation

A 71-year-old female presented with a past medical history significant for coronary artery disease with several angioplasties and eight drug eluting stents from 1995-2018, hyperlipidemia, hypertension, and CLL that was last treated with rituximab two weeks prior to admission. The patient is compliant with prasugrel but was temporarily paused due to recent chemotherapy. The patient presented to the ED after experiencing chest pain on exertion that was temporarily relieved with sublingual nitroglycerin. However, chest pain evolved and was eventually refractory to sublingual nitroglycerin. The pain was largely midsternal, non-radiating, and rated 10/10 on pain scale. There was no associated shortness of breath, palpitations or orthopnea. Of note, patient’s last diagnostic heart catheterization a year prior to presentation demonstrated patent vessels and stents.

**Figure 1:**
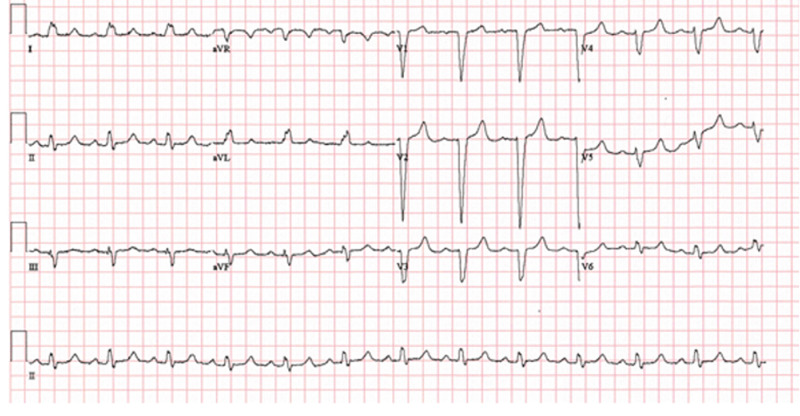
Electrocardiogram of patient upon admission, endorsing the underlying left bundle branch block as well as a prolonged PR interval.

Hematologic data was significant for a white blood cell count (WBC) of 224 k/uL with a 93.9% lymphocytic predominance. There was a normocytic anemia with a hemoglobin (Hg) of 9.9 g/dL and mean corpuscular volume (MCV) of 86.3 fL. Platelet level was 173 k/uL. With the exception of the WBC, all of these values are at or near patient's relative baseline within the last year. Troponins peaked at 0.53 ng/ml on the day of admission. Regarding imaging, chest X-ray was negative for acute cardiopulmonary disease. Electrocardiogram (EKG), as seen in Figure 1, was significant for left axis deviation and a left bundle branch block (LBBB) with PR prolongation; however, these findings were unchanged compared to the most recent EKG over a year ago.

After obtaining informed consent, the patient was transferred to the Cardiac Cath Lab for urgent catheterization. The patient was anticoagulated with intravenous (IV) bivalirudin. The patient was initially given a 12-mg bolus of bivalirudin followed by an infusion at 137.5 mcg for the duration of the catheterization. A transfemoral approach was used for arterial access. A 6-French sheath and pigtail catheter were used to cross the aortic valve and evaluate the left ventricular pressures. A 6-French JL4 catheter was used for left and right coronary angiography. Cardiac catheterization findings were significant for a normal systolic left ventricular function ejection fraction (EF) of 60-65%. The left main artery was patent and showed no signs of coronary artery disease. The left anterior descending (LAD) had 30% stenosis in the proximal and mid LAD with patent stents. The left circumflex (LCFX) had 30% proximal LCFX stenosis with a patent stent in the OM branch. The mid right coronary artery had 80% segmental stenosis and was ultimately targeted for drug eluting stent (DES) deployment. The right coronary artery (RCA) was engaged with a 6F FR4 guide and a 0.014” BMW wire. A 2.5 x 15 mm balloon was placed along the lesion and inflated to 8 atm. A follow-up angiogram demonstrated improvement in stenosis without complications. The lesion was stented with a 3.0 x 34 mm Onyx DES and inflated to 18 atm (FLD 3.25 mm). Follow-up angiography confirmed excellent flow without signs of residual stenosis. There was no dissection or compromise in arterial wall integrity. TIMI III flow was restored, and the procedure was concluded. Bivalirudin was discontinued and the patient was started on dual antiplatelet therapy with aspirin 81 mg and prasugrel 60 mg.

The patient was admitted to the coronary care unit (CCU) for post-PCI recovery and monitoring. The patient’s laboratory data on the day of discharge was significant for a WBC count of 153 k/uL, Hg of 10.6 g/dL, platelets of 153 k/uL and a chemistry panel significant for improved Cr from 1.41 mg/dl to 1.27 mg/dl. EKG was still significant for left axis deviation in setting of LBBB but was unchanged compared to prior studies. Post-operative echo was significant for an EF of 55-60% without evidence of wall motion abnormalities. Of note, there was mild mitral regurgitation and physiologic pericardial effusion but no tamponade pathology. The patient was hemodynamically and vitally stable, asymptomatic without chest pain, breathing well on room air and showing no signs of bleeding or bruising.

## Discussion

Cardiac catheterization is an invasive procedure that inherently increases the risk of clot formation, thus necessitating adequate anticoagulation. Unfractionated heparin is the most commonly used anticoagulant during cardiac catherization. However, the data on the ideal drug of choice in patients with leukemia is limited. In part, the ideal candidate is not clear because the mechanism of thrombosis varies in patients with leukemia versus those without. Although the cornerstone of coagulability is best summarized by Virchow’s triad, the mechanism of thrombosis varies significantly between different malignancies. Heparinase, a beta-endoglucouronidase, is an enzyme that cleaves heparin throughout the extracellular matrix. Heparinase is physiologically found in platelets, neutrophils and monocytes and been demonstrated to worsen inflammation and progression of cancerous diseases [7]. This pathway may demonstrate why heparins are not the ideal anticoagulant of choice in patients with myelogenous leukemias and why bypassing the pathway with the use of direct thrombin inhibitors may be effective. There is insufficient data available to help explain unique pathways in thrombosis formation amongst patients in CLL that would advise for use of one anticoagulant versus another. We present the most commonly used anticoagulants in patients undergoing PCI as well as an explanation as to why we believe bivalirudin may be a promising, but not ideal, agent amongst patients with CLL. As will be discussed, bivalirudin's greatest merit is that it has a lower risk of bleeding but higher risk of in-stent thrombosis when compared to heparin.

UF-heparin is the most widely used and characterized anticoagulant in PCI. The greatest benefit of UF-heparin is its ease of use, reversibility and that it can be easily monitored at bedside. However, UFH use alone is limited in that while it minimizes the chances of new clots from forming, it has no effect against thrombin that is already clot bound. This requires the concomitant use of a glycoprotein inhibitor or clopidogrel to reduce periprocedural ischemic complications [8]. Another notorious concern when using heparin for prolonged periods of time is the risk of developing heparin-induced thrombocytopenia. Although a single PCI would carry a small risk of heparin-induced thrombocytopenia (HIT), we must consider that patients in such a demographic are likely to have repeated exposures to heparin. The development of thrombocytopenia in setting of PCI carries a much higher risk of morbidity and mortality [9].

The limitations of heparin have prompted for a search of better alternatives. Low molecular weight heparins (LMWH) gained traction because of their better activity specifically against Factor Xa rather than thrombin. Since factor Xa catalyzes the formation of thrombin, the reasoning was that aggressively inhibiting factor Xa would indirectly solve the problem posed by high levels of thrombin. Enoxaparin, the flagship of LMWH, has been studied extensively. The STEEPLE trial compared intravenous variants of 0.5 mg/kg and 0.75 mg/kg enoxaparin versus UFH [10] while the SYNERGY trial evaluated the efficacy versus UFH in 10,027 high risk patients with acute coronary syndrome (ACS) [11]. Both studies demonstrated that Enoxaparin was statistically non-inferior versus UFH. A series of meta-analysis also went on to confirm that LMWH is non-inferior UFH in setting of elective PCI [12]. The biggest limitation of LMWH is a lack of bedside monitoring. Additionally, given that it is in the class of heparins there is always a risk, though smaller compared to heparin, of inducing HIT.

Fondaparinux is another well-known anticoagulant. It is an indirect inhibitor of Factor Xa with no direct effect on thrombin. The intrinsic downstream effect in the clotting cascade makes it a good agent for preventing clots, however, since it does not bind thrombin its use is limited in preventing catheter-related thrombosis due to thrombin. Additionally, it is an irreversible drug with a half-life of 17-21 hours thus making it difficult to monitor at bedside as well as to be reversed. Fondaparinux was proven to be statistically noninferior versus UFH in setting of non-STEMI acute coronary syndrome (ACS) as based on the OASIS 5 trial. The trial demonstrated that the 9-day composite of death, myocardial infarction (MI), and refractory ischemia was non-inferior but was statistically superior in terms of 30-day major bleeding and 30/180-day mortality benefit versus UFH [8]. However, the OASIS 5 trial then went on to demonstrate that amongst the 6,238 patients undergoing PCI, those that received Fondaparinux had a significant reduction in bleeding at the 9-day mark but also had significantly higher rate of catheter-related thrombosis [13].

Finally, one of the most promising anticoagulants are the direct thrombin inhibitors (DTIs). DTIs do not rely on antithrombin or factor Xa on their anticoagulant properties and are directly active against thrombin itself. Bivalirudin is one of the most widely used DTIs with an irreversible binding to thrombin and a half-life of approximately 25 minutes. Bivalirudin was first studied in the Bivalirudin Angioplasty Trial that evaluated 4098 patients undergoing balloon angioplasty in setting of unstable or post-infarction angina. Bivalirudin demonstrated a reduced risk of bleeding versus UFH (3.8% vs 9.8%; P < 0.001) [14]. Following the results of this trial, over 25,000 patients were studied in trials comparing bivalirudin versus UFH and Enoxaparin in setting of elective PCI vs PCI due to acute STEMI. The ISAR-REACT 4 trial compared bivalirudin to UFH in patients undergoing elective PCI. This trial demonstrated risk of major bleeding was reduced with bivalirudin [15]. Interestingly, the most recent major study involving bivalirudin was the HORIZONS AMI trial that compared bivalirudin to UFH + GPI in settings of primary PCI for STEMI. This study included patients that were 18 years of age or older who presented within 12 hours of symptom onset and were found to have an ST-segment elevation of at least 1 mm in two or more contiguous leads, a new left bundle branch block, or a posterior myocardial infarction. An extensive list of exclusion criteria was provided, however, none of which pertained to our patient. Bivalirudin emerged as the leader with a reduced rate of 30-day major rebleeding (4.9% vs 8.3%; P < 0.001). The rate of stroke, death, MI were nearly identical. The interesting difference stemmed to 24-hour stent thrombosis, which demonstrated that bivalirudin was associated with higher risk vs UFH-GPI (1.3% vs 0.3% PP < 0.001) [16]. This claim was further supported by a systematic review focusing on clinical trials that compared bivalirudin to heparin from January 2000 to December 2017 in over 53,000 patients. Narayanan et al. reported that bivalirudin was associated with less major bleeding complications, however, there was an increased incidence of in-stent thrombosis [17]. Although bivalirudin has this shortcoming, there is data on post-PCI agents that have favorable IST outcomes. New data has emerged that suggests Prasugrel is superior to Clopidogrel in terms of risk of stent restenosis [18]. Our patient received Prasugrel pre- and post-procedure, thus mitigating the risk of stent thrombosis cited by the HORIZONS trial.

## Conclusions

As previously discussed, we believe that bivalirudin may be a promising agent and the most reliable for use in high-risk populations such as patients with an inherently high risk of clotting or bleeding. Unfractionated heparin, while widely used, is not favorable because of the higher risk of bleeding versus bivalirudin as demonstrated by the Bivalirudin Angioplasty Trial. Fondaparinux is also not an ideal choice because although it proved to have a lower risk of bleeding, there was a significantly higher risk of catheter thrombosis versus UFH. Therefore, we believe that our case report can contribute to the growing body of evidence that bivalirudin may be a favorable choice for anticoagulation during cardiac catheterization for patients with a high risk of bleeding.

## References

[1] Xu Z, Sun Y, Wei Z, Jiang J, Xu J, Liu P (2020). Suppression of CXCL-1 could restore necroptotic pathway in chronic lymphocytic leukemia. Onco Targets Ther.

[2] Wang TY, Magid DJ, Ting HH, Li S, Alexander KP, Roe MT, Peterson ED (2014). The quality of antiplatelet and anticoagulant medication administration among ST-segment elevation myocardial infarction patients transferred for primary percutaneous coronary intervention. Am Heart J.

[3] Krolick MA (2006). Successful percutaneous coronary intervention using bivalirudin in a patient with chronic lymphocytic leukemia and thrombocytopenia. Eur J Haematol.

[4] O’Gara PT, Kushner FG, Ascheim DD (2013). 2013 ACCF/AHA guideline for the management of ST-elevation myocardial infarction: a report of the American College of Cardiology Foundation/American Heart Association Task Force on Practice Guidelines. Circulation.

[5] Steg PG, James SK, Atar D (2012). ESC Guidelines for the management of acute myocardial infarction in patients presenting with ST-segment elevation: the task force on the management of ST-segment elevation acute myocardial infarction of the European Society of Cardiology (ESC). Eur Heart J.

[6] Potts J, Mohamed MO, Lopez Mattei JC (2020). Percutaneous coronary intervention and in-hospital outcomes in patients with leukemia: a nationwide analysis. Catheter Cardiovasc Interv.

[7] Horowitz NA, Brenner B (2020). Thrombosis in hematological malignancies: mechanisms and implications. Thromb Res.

[8] Kastrati A, Mehilli J, Neumann FJ (2006). Abciximab in patients with acute coronary syndromes undergoing percutaneous coronary intervention after clopidogrel pretreatment: the ISAR-REACT 2 randomized trial. JAMA.

[9] Wang TY, Ou FS, Roe MT, Harrington RA, Ohman EM, Gibler WB, Peterson ED (2009). Incidence and prognostic significance of thrombocytopenia developed during acute coronary syndrome in contemporary clinical practice. Circulation.

[10] Montalescot G, White HD, Gallo R (2006). Enoxaparin versus unfractionated heparin in elective percutaneous coronary intervention. N Engl J Med.

[11] Ferguson JJ, Califf RM, Antman EM (2004). Enoxaparin vs unfractionated heparin in high-risk patients with non-ST-segment elevation acute coronary syndromes managed with an intended early invasive strategy: primary results of the SYNERGY randomized trial. JAMA.

[12] Dumaine R, Borentain M, Bertel O (2007). Intravenous low-molecular-weight heparins compared with unfractionated heparin in percutaneous coronary intervention: quantitative review of randomized trials. Arch Intern Med.

[13] Mehta SR, Granger CB, Eikelboom JW (2007). Efficacy and safety of fondaparinux versus enoxaparin in patients with acute coronary syndromes undergoing percutaneous coronary intervention: results from the OASIS-5 trial. J Am Coll Cardiol.

[14] Bittl JA, Strony J, Brinker JA (1995). Treatment with bivalirudin (Hirulog) as compared with heparin during coronary angioplasty for unstable or postinfarction angina. N Engl J Med.

[15] Kastrati A, Neumann FJ, Mehilli J (2008). Bivalirudin versus unfractionated heparin during percutaneous coronary intervention. N Engl J Med.

[16] Stone GW, Witzenbichler B, Guagliumi G (2008). Bivalirudin during primary PCI in acute myocardial infarction. N Engl J Med.

[17] Narayanan M, Anugula D, Gujjula NR (2018). Bivalirudin versus heparin in percutaneous coronary intervention—a systematic review and meta-analysis of randomized trials stratified by adjunctive glycoprotein IIb/IIIa strategy. J Thorac Dis.

[18] Norgard NB, Abu-Fadel M (2009). Comparison of prasugrel and clopidogrel in patients with acute coronary syndrome undergoing percutaneous coronary intervention. Vasc Health Risk Manag.

